# A One Health Perspective on Camel Meat Hygiene and Zoonoses: Insights from a Decade of Research in the Middle East

**DOI:** 10.3390/vetsci11080344

**Published:** 2024-07-29

**Authors:** Mohamed-Yousif Ibrahim Mohamed, Glindya Bhagya Lakshmi, Hamidreza Sodagari, Ihab Habib

**Affiliations:** 1 Veterinary Public Health Research Laboratory, Department of Veterinary Medicine, College of Agriculture and Veterinary Medicine, United Arab Emirates University, Al Ain P.O. Box 1555, United Arab Emirates; mohamed-yousif-i@uaeu.ac.ae (M.-Y.I.M.); glindya_l@uaeu.ac.ae (G.B.L.); 2 ASPIRE Research Institute for Food Security in the Drylands (ARIFSID), United Arab Emirates University, Al Ain P.O. Box 1555, United Arab Emirates; 3 Department of Pathobiology, College of Veterinary Medicine, University of Illinois Urbana-Champaign, Urbana, IL 61802, USA; hr.sodagari@gmail.com

**Keywords:** camels, zoonotic diseases, food safety, risk analysis

## Abstract

**Simple Summary:**

This review explores the safety of camel meat and the diseases that camels can transmit to humans in the Middle East conducted over the past ten years, emphasizing the need for a One Health approach. An examination of recent studies indicated significant issues with pathogens, including antibiotic-resistant bacteria and contamination with heavy metals and pesticides. The review also highlighted the ongoing risk of diseases like Middle East respiratory syndrome coronavirus (MERS-CoV) and other zoonoses. Findings from this review call for more robust food safety measures and increased cooperation among veterinary and public health authorities to ensure the safety of camel meat and protect public health.

**Abstract:**

The purpose of this review was to investigatethe microbial and chemical safety of camel meat and the zoonotic diseases associated with camels in the Middle East over the past decade, emphasizing the crucial role of a One Health approach. By systematically analyzing recent studies (in the past decade, from 2014), we assessed pathogen prevalence, contamination with heavy metals and pesticide residues, and the impact of zoonotic diseases like Middle East respiratory syndrome coronavirus (MERS-CoV). The findings revealed significant variability in pathogen prevalence, with the frequent detection of traditional foodborne pathogens (e.g., *Salmonella* and *E. coli O157*), as well as antibiotic-resistant strains like methicillin-resistant and vancomycin-resistant *Staphylococcus aureus* and extended-spectrum β-lactamase (ESBL)-producing *E. coli*, underscoring the need for stringent antibiotic use policies and robust food safety measures. Additionally, the review highlighted substantial contamination of camel meat with heavy metals and pesticide residues, posing significant public health concerns that necessitate stringent regulatory measures and regular monitoring. The persistent occurrence of zoonotic diseases, particularly MERS-CoV, along with other threats like trypanosomiasis, brucellosis, and *Clostridium perfringens*, emphasizes the importance of strengthening ongoing surveillance. Enhancing investment in diagnostic infrastructures, training programs, and planning capabilities is crucial to address these issues at the camel–human interface in the Middle East. Adopting a One Health perspective is vital to ensuring the safety and quality of camel meat and managing zoonotic risks effectively to ultimately safeguard public health and promote sustainable livestock practices.

## 1. Introduction

Camels are known as the champions of deserts and highlands, thriving in regions with challenging climates. Camelidae includes two subfamilies: Camelinae (Old World camelids) and Lamini (New World camelids). The two main species of Old World camels are, as follows: (i) the Dromedary camel (*Camelus dromedarius*), found in the hot, arid areas of the Middle East and Africa; and (ii) the Bactrian camel (*Camelus bactrianus*), the largest camel species with two humps on its back, living in northwestern China and southwestern Mongolia. New World camelids are found in South America. The four species of New World camelids are, as follows: (iii) the llama (*Lama glama*); (iv) the alpaca (*Lama pacos*); (v) the guanaco (*Lama guanaco*); and (vi) the vicuña (*Vicugna vicugna*) [1,2].

Camels have been integral to the history and culture of the Middle East, where they have been closely connected with human societies for centuries [3]. Camels are considered multipurpose animals, offering meat, milk, wool, hair, and dung. Female camels are mainly utilized for their milk, while male camels are employed for transportation and draft work [4]. In some Arabian countries, camel meat is the preferred choice for many traditional dishes and is often favored over other types of red meats. Most of the meat comes from male and female camels that are seven years old or older and are primarily raised for milk, racing, and transportation rather than for meat production alone [5,6,7]. Typically, a dromedary camel carcass can produce between 125 kg and 400 kg or more of meat, with the amount varying depending on the breed, sex, and age at slaughter [8]. In addition to its important contribution to transportation, draft work, and food security in the Middle East, the camel plays a vital role as a sport animal in the region [9]. Camel racing, deeply rooted in Bedouin ancestral culture, is a prominent traditional sport in many Arab countries and is known for its profitability and efficient organization. It is of significant importance as a traditional activity and is an essential aspect of the animal agribusiness within the Gulf Cooperation Council (GCC) countries, especially in the United Arab Emirates (UAE), Saudi Arabia, and Qatar [8,9].

The United Nations has designated 2024 as the International Year of Camelids (IYC 2024) to emphasize camelids’ crucial role in combating hunger by contributing to food security, nutrition, and economic development in areas where other livestock cannot thrive [10]. Therefore, this review focuses on exploring, as follows: (a) the microbial and chemical safety of camel meat, and (b) camel-associated zoonotic diseases across Middle Eastern countries, where the camel plays a significant role in food and animal agriculture. This review will focus on the advances in regional research generated over the past decade (from 2014 onward). 

## 2. Camel Meat Production and Supply Chain in the Middle East

Globally, camel husbandry has garnered increasing social and economic interest over the past three decades due to the international importance of camels as a sustainable livestock species [11]. Due to rising food demand, urbanization, and concerns about deforestation from open grazing, camel rearing has transitioned towards intensive or semi-intensive farming methods [12]. Dromedary camels are considered one of the most suitable breeds for meat production in arid land due to their ability to endure adverse climatic conditions, which sets them apart from other breeds [13]. The camel meat industry operates on a regional scale, leading to the export of live camels rather than carcasses. The nutritional quality of camel meat varies with age, breed, and muscle type [13,14]. Camel meat is considered a healthy option because it contains less fat and cholesterol and is rich in essential fatty acids such as omega-3 fatty acids [14,15,16,17,18]. It is also a good source of minerals, vitamins, and bioactive compounds compared to meat from other animals [7,18,19,20]. The consumption of camel meat has been linked to a likely reduction in hypertension and hypersensitivity (allergic reactions or intolerances) [6,20,21].

Camel productivity and profitability have historically been hindered by factors such as limited sales opportunities, restricted slaughter practices, low yields, and a high mortality rate [22,23,24]. In the Middle East, farmers typically pay minimal attention to production costs. The primary reason for this is that a significant portion of the expenses associated with establishing and maintaining camel farms is covered/subsidized by government support [11]. Consequently, many farm owners do not view camel farming as their primary occupation [11].

Producers, processors, and retailers of camel meat must stay informed about, and comply with, relevant hygiene standards and regulations to ensure the safety and quality of the product [25,26,27,28]. In the following sections of this review, we will examine recent studies exploring biological hazards linked to camel meat consumption. We will also draw insights from emerging findings on the chemical contaminants found in camel meat, elaborating on data from published studies in the Middle East from 2014 onward.

## 3. Methodological Framework and Data Acquisition

An extensive literature search was performed using multiple databases. The primary databases utilized included PubMed and Google Scholar, which were chosen for their broad coverage of biomedical and scientific literature. We excluded non-peer-reviewed sources, such as Letters to the Editor, opinion pieces, and anecdotal reports, to ensure the reliability and scientific rigor of the information presented. Some of the data on zoonotic outbreaks were sourced from international organizations (e.g., the World Organization for Animal Health (WOAH) World Animal Health Information System (WAHIS), and the World Health Organization (WHO) Global Health Observatory (GHO) and Disease Outbreak News (DON) reporting systems. The search focused on articles published in English over the past decade (from 2014) to ensure the inclusion of the most recent and relevant research. The search strategy involved using Boolean operators to combine keywords, enhancing the retrieval of pertinent articles [29]. The search terms were: ((“camel meat” OR “camelmeat” OR “camel” AND “meat”) AND (hygiene OR sanitation OR “microbial quality” OR “microbial hazard” OR “chemical quality” OR “chemical hazard” OR “food safety”)) AND (zoonoses OR “zoonotic diseases” OR “infectious diseases” OR “camel diseases”) AND (“One Health” OR “interdisciplinary approach” OR “human-animal-environment interaction”) AND (“Middle East” OR “Arabian Peninsula” OR “Near East” OR “North Africa”).

**Figure 1:**
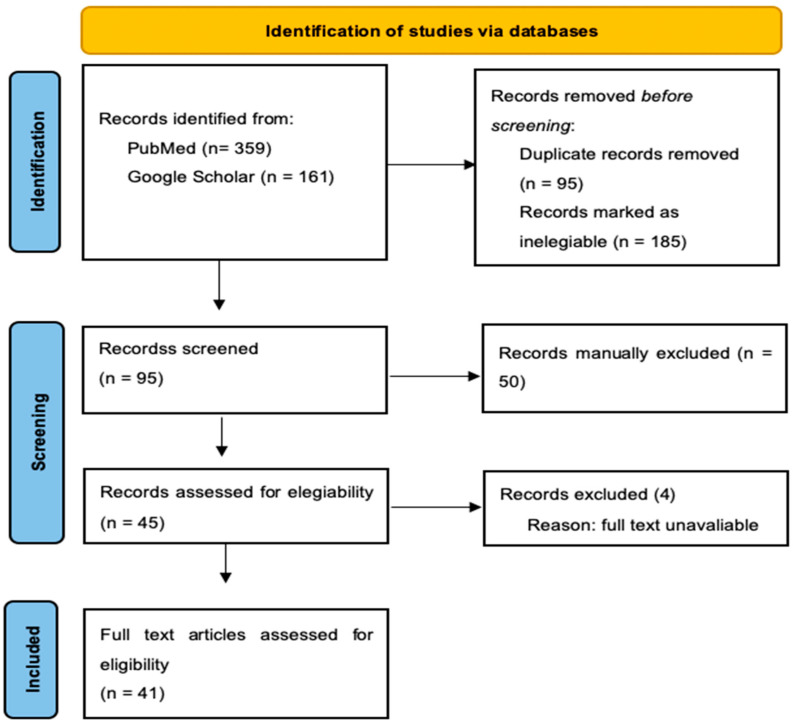
PRISMA 2020 flow diagram for updated reviews in the database search.

Additionally, variations and synonyms of these terms were included to ensure that no relevant studies were overlooked. Inclusion criteria were established to filter the articles for relevance. Only studies that provided insights into microbial and chemical safety and zoonotic diseases in camel meat were considered. Exclusion criteria were also defined, eliminating studies not directly related to camel meat hygiene or those published in languages other than English. The initial search yielded a large number of articles (Figure 1), which were then subjected to a rigorous screening process by two of the co-authors. Titles and abstracts were reviewed to assess their relevance, followed by a full-text review of the selected articles. Data extraction was performed systematically, focusing on study design, methodology, findings, and hazards related to camel meat hygiene [30]. Figure 1 represents the systematic approach to the inclusion and exclusion of articles.

## 4. Microbial Hazards of Camel Meat in the Middle Eastern Countries over the Past Decade

Food safety is an international issue, and according to the WHO, the Middle East and North Africa (MENA) ranks third in the highest estimated burden of foodborne diseases per population [31]. The microbial safety of camel meat is crucial for public health, as the consumption of contaminated meat can contribute to the overall burden of foodborne disease in the region. 

The findings in Table 1, from articles found in the database search, provide insights into the potential hazards associated with camel meat consumption in the Middle East over the past decade. The available data reveal significant variability in pathogen prevalence across different countries (Table 1), indicating differing standards in meat handling, hygiene practices, and detection capabilities. The studies utilized a range of detection methods, including conventional cultural techniques, as well as various molecular techniques. Studies conducted in Egypt, Iran, Libya, and Saudi Arabia between 2014 and 2024 highlight the presence of various pathogens in camel meat and related products (Table 1). In Egypt, research conducted in 2019 identified *Pseudomonas aeruginosa* and *P. fluorescens* in 10% of meat samples, while Vancomycin-resistant *Staphylococcus aureus* (VRSA) was found in 14.5% of samples [32,33]. Further studies in 2023 detected *Escherichia coli* O157 in 9.1% of meat samples and extended-spectrum β-lactamase (ESBL)-producing *E. coli* in 8% of the samples [34,35]. By 2024, *Salmonella* spp. had a prevalence of 8% in camel meat samples, with multiple serotypes showing a 20% presence in meat, liver, and kidney samples [36]. In Iran, *Acinetobacter baumanii* was identified in 2.26% of meat samples in 2019, and *Staphylococcus aureus* had a 4% prevalence in samples in 2020 [37,38]. A study in Libya reported an intriguing prevalence of *Vibrio parahaemolyticus* in camel meat samples, at 33.33% in 2016 [39].

Saudi Arabia’s studies indicated the prevalence of varyious pathogens over the years (Table 1). In 2015, *E. coli* O157 was found in 2.4% of fecal samples and 2.9% of hides, with *Salmonella* showing a prevalence of 23.2% in the feces and 67.6% in the hides of camels [42]. In 2016, methicillin-resistant *Staphylococcus aureus* (MRSA) was detected in 20% of meat samples [43]. A report in the year 2020 pointed to the detection of *Listeria monocytogenes* in 16% of camel meat samples, and other *Listeria* species were also detected [44]. ESBL-producing *E. coli* had an 11.3% prevalence in minced camel meat [45]. In 2021, *Clostridium perfringens* was present in 14% of minced camel meat, and Metallo-β-lactamase (MβL)-producing *Pseudomonas* spp. showed a high prevalence of 55% in the screened meat from camels [46,47].

The recurring detection of traditional foodborne pathogens (e.g., *Salmonella*, *Listeria monocytogenes*, and *E. coli O157*), as well as antibiotic-resistant strains, like MRAS, VRSA, and ESBL-producing *E. coli*, underscores the urgent need for stringent antibiotic use policies and foodborne pathogen management strategies. Overall, the summary of results in Table 1 indicates substantial challenges in ensuring the microbial safety of camel meat, with potential public health implications. These findings highlight the need for enhanced food safety measures, improved hygienic practices, and robust surveillance systems targeting camel meat consumed in the Middle East.

## 5. Chemical Hazards of Camel Meat in the Middle Eastern Countries over the Past Decade

While camels serve as a complementary source of red meat in certain Middle Eastern nations, research focusing on heavy metals contamination across the region has predominantly centered on the meat and products of cattle, sheep, and goats, with few investigations conducted on camel meat (Table 2). The consumption of meat contaminated with chemical residues and contaminants could affect human health and poses significant public health challenges [49]. The bioaccumulation and biomagnification of heavy metals in camels occur when they are exposed to environmental pollution when, for example, they freely graze in contaminated areas or consume polluted water [50]. 

Table 2 elaborates on the status of chemical residues and contaminants in camel meat and meat products in the Middle Eastern region over the past decade, based on evidence from articles found in the database search. The results reveal significant contamination (above certain levels stipulated by national standards) with heavy metals, pesticide residues, and drug residues, highlighting critical concerns for food safety. Notably, high contamination levels were reported in several countries (Table 2). In Egypt, a 2015 study identified the presence of heavy metals, such as lead (Pb), cadmium (Cd), copper (Cu), and zinc (Zn), in muscle, serum, lungs, liver, and kidney samples using atomic absorption spectrophotometry (AAS) [51]. In the same year, another study detected oxytetracycline and heavy metals in muscles, kidneys, and liver [52]. El Gareeb et al. [53] reported the presence of various antibiotic residues, including enrofloxacin, ciprofloxacin, and tetracycline derivatives, in muscle, kidney, and liver samples, detected using LC-MS/MS (Table 2).

In Iraq, studies conducted in 2020 and 2021 identified heavy metals, including arsenic (As), chromium (Cr), and cobalt (Co), in muscle, kidney, and liver samples from camels using inductively coupled plasma–optical emission spectrometry (ICP–OES) [54,55]. Similarly, high levels of heavy metals were reported in Saudi Arabia, with studies from 2015 to 2024 consistently detecting Pb, Cd, Hg, As, and Cr in various camel tissues, including in the muscles and liver [53,59,60,62,63,64]. In 2015, a study in Saudi Arabia also reported the detection of pesticide residues, such as DDT and its metabolites, in the muscles and liver of camels, using Gas Chromatography/Mass Spectrometry (GC/MS) [59].

Detecting heavy metals, especially lead and cadmium, alongside persistent organic pollutants, like DDT and its metabolites, in edible camel tissues underscores a significant public health concern due to the potential for toxic accumulation in humans through the food chain. These contaminants can lead to severe health issues, including neurological, reproductive, and developmental problems [49,50]. Antibiotic residues in camel meat further complicate the issue, raising questions about the misuse of veterinary drugs and the potential for antibiotic resistance. These findings highlight the need for stringent regulatory measures and regular monitoring efforts to ensure the safety of camel meat with regard to chemical residues and contaminants. In Middle Eastern countries, where camel meat is more commonly consumed, future studies must focus on the long-term health implications, the sources of contamination, and effective mitigation strategies. Enhanced analytical methods and comprehensive risk assessments of chemical residues and contaminants in camel meat are essential to address these food safety challenges.

## 6. Zoonoses in Camel Populations in the Middle Eastern Countries

One of the most widely known zoonotic diseases connected with camels over the past decade is the Middle East respiratory syndrome coronavirus (MERS-CoV) [65]. Recognizing the range of camel-related zoonotic diseases is essential for carrying out reliable control actions and reducing health threats. Zoonotic infections can spread from camels to humans via direct contact with infected animals, contaminated food products, or exposure to contaminated environments [65,66]. For instance, traditional camel management practices, slaughtering approaches, processing aspects, and hygienic procedures are among the key factors influencing the course of these zoonotic diseases [67]. Implementing appropriate control and prevention processes for zoonotic diseases related to camels is contingent upon the One Health concept, which embraces system thinking and addresses diseases at the animal–human–environment interfaces [68,69]. Techniques that include camel vaccination, the introduction of biosecurity systems in farms and retail markets, the recommendation of hygienic food handling practices, and public health education can be employed to minimize the risk of camel-associated zoonotic transmission [70].

In the Middle East, there has been a tangible improvement in the surveillance and monitoring of significant zoonotic risks in camels, notably MERS-CoV and brucellosis; however, there are still significant gaps [65]. A critical gap is the lack of an operational One Health surveillance system that could integrate data from both human and animal health sectors [23]. Some countries, especially in the GCC region, have established surveillance programs for specific zoonotic diseases like MERS-CoV or brucellosis. However, these initiatives are isolated, and efficient system thinking and transparent data-sharing must be further considered [71]. One of the gaps is also in the diagnostic methods used to identify zoonotic pathogens in the camel population. More sensitive and specific methods are required, especially for rapidly detecting multiple emerging zoonotic diseases [65]. Moreover, applying new technologies for pathogen identification in some Middle Eastern countries, such as next-generation sequencing, is not equally accessible due to economic challenges; hence, accurate monitoring is hindered. There is a need, across the region, to invest more in diagnostic infrastructures, planning capabilities, and training programs to improve capacities and strengthen surveillance systems [72]. Baseline studies on camel zoonoses are lacking in many Middle Eastern countries; as such, there is a gap in knowledge on transmission routes and contributing environmental factors. This knowledge is in high demand to guide evidence-based policies and practices in preventing and controlling zoonotic diseases.

Table 3 provides a detailed review of human cases of camel-origin zoonotic diseases in the GCC and north African countries over the past decade (2014 and onward). MERS-CoV remains a persistent zoonotic challenge issue in several countries across the regions (Table 3). For instance, multiple outbreaks in humans were reported in Saudi Arabia over the past decade (Table 3). The most significant outbreak in 2014–2015 involved 566 human cases, all of which required hospitalization and resulted in 69 deaths [72]. The prevalence of direct contact with camels as the primary transmission route underscores the critical need for stringent control measures in camel handling and interactions (Table 3).

Similarly, the UAE reported significant outbreaks in humans between 2017 and 2022, with 14 cases requiring hospitalization [71]. Oman and Qatar also experienced substantial MERS-CoV activity, with Oman recording 18 human cases from 2017 to 2022, all requiring hospitalization [71], and Qatar reported 16 human cases from 2014 to 2016, all requiring hospitalization [72]. On the other hand, Algeria and Bahrain experienced fewer, but a still significant amount of, MERS-CoV human cases, with Algeria documenting an outbreak in 2014 that resulted in two deaths [72], while Bahrain had an outbreak in 2016 with 1 death [71]. On the other hand, Egypt reported Trypanosomiasis and *Clostridium perfringens* outbreaks linked to camel meat consumption, highlighting the potential for non-traditional zoonotic threats beyond MERS-CoV (Table 3).

The recurrence of MERS-CoV outbreaks across the Middle East over the past decade emphasizes the zoonotic risks associated with camel–human interactions across the region. This consistent pattern necessitates ongoing surveillance, improved One Health strategies, and better veterinary preparedness to mitigate these risks.

## 7. Conclusions

Over the past decade, significant research endeavors have been made to understand camel meat’s microbial and chemical safety and the zoonotic risk in the Middle East; however, there is considerable variability in pathogen surveillance efforts across different countries. The persistent detection of antibiotic-resistant strains underscores the urgency for stringent antibiotic use policies and robust food safety measures. Enhanced hygienic practices and surveillance systems are crucial to mitigate these microbial risks and protect public health. The findings from this review also highlight a significant contamination of camel meat with heavy metals, pesticide residues, and antibiotic residues, posing a substantial public health concern. MERS-CoV continues to be a persistent public health issue in the region, with numerous outbreaks reported.

We acknowledge that English might not be the predominant publishing language in some Middle Eastern countries, where Arabic, French, and Farsi are used for local scientific publications. Consequently, limiting our search to English-language publications may have restricted our review findings. However, we chose to focus on English to ensure accessibility and consistency in the review process and to utilize widely accessible databases. Future research could expand to include additional languages for a more comprehensive overview of the topic.

Baseline studies on camel zoonoses are needed to fill knowledge gaps on transmission routes and contributing environmental factors. Collaborative efforts among governments, researchers, and veterinary and public health authorities are crucial to the development of evidence-based policies and practices to control food safety hazards and prevent the zoonotic diseases associated with camels.

## Figures and Tables

**Table 1:** Overview of research on microbial hazards of camel meat in some Middle Eastern countries published between 2014–2024.

Country	Year	Pathogen(s)	Source(s)	Sample Size	Prevalence (%)	Detection Method	Reference
Egypt	2019	*Pseudomonas aeruginosa*	Meat	100	8.0	Biochemical identification by VITEK 2 and PCR confirmation	[32]
		*Pseudomonas fluorescens*			2.0		
Egypt	2019	Vancomycin-resistant *Staphylococcus aureus* (VRSA)	Meat	200	14.5	Culture-based detection, biochemical identification, and PCR confirmation	[33]
Egypt	2023	*Escherichia coli* O157:H7	Meat	110	9.1	Culture-based detection, biochemical and serological identification, and PCR confirmation	[34]
		*Escherichia coli* O55:H7			32.0		
Egypt	2023	Extended Spectrum β-lactamase (ESBL) producing *Escherichia coli*	Meat	50	8.0	Culture-based detection, biochemical identification, and PCR confirmation. Double-Disc Synergy Test (DDST) for ESBL characterization	[35]
Egypt	2024	*Salmonella* Enteritidis *Salmonella* Typhimurium *Salmonella* Virchow *Salmonella* Apeyeme	Meat, liver, kidney	60	20.0	Culture-based detection, biochemical and serological identification, and PCR confirmation	[36]
Iran	2019	*Acinetobacter baumanii*	Meat	95	2.3	Culture-based detection and PCR confirmation	[37]
Iran	2020	*Staphylococcus aureus*	Meat	100	4.0	Culture-based detection, biochemical identification, and PCR confirmation	[38]
Libya	2016	*Vibrio parahaemolyticus*	Meat	9	33.3	Culture-based detection, PCR confirmation	[39]
Egypt	2024	*Salmonella* spp.	Meat	100	8.0	Culture-based detection, biochemical identification, and PCR confirmation	[40]
		*Staphylococcus aureus*			24.0		
		*Escherichia coli*			14.0		
Egypt	2024	*Escherichia coli* strains O17:H18; O128:H2, O119: H6, O103:H4, O145, O121:H7	Meat	60	10.0	Culture-based detection, serological identification, and PCR confirmation	[41]
			Liver		30.0		
			Kidney		5.0		
Saudi Arabia	2015	*Escherichia coli* O157	Feces	206	2.4	Culture-based detection, biochemical identification, and PCR confirmation	[42]
			Hides		2.9		
		*Salmonella* spp.	Feces		23.2		
			Hides		67.6		
Saudi Arabia	2016	Methicillin-resistant *Staphylococcus aureus* (MRSA)	Meat	24	20.0	Culture-based detection, biochemical identification, molecular characterization by StaphyType DNA microarray technology	[43]
		Methicillin-susceptible *Staphylococcus aureus* (MSSA)			28.0		
Saudi Arabia	2020	*Listeria monocytogenes*	Meat	50	16.0	Culture-based detection, biochemical identification and PCR confirmation	[44]
		*Listeria seeligeri*			6.0		
		*Listeria innocua*			2.0		
		*Listeria welshimeri*			2.0		
		*Listeria grayi*			1.0		
Saudi Arabia	2020	Extended Spectrum β-lactamases (ESBL) producing *Escherichia coli*	Minced meat	150	11.3	Culture-based detection, biochemical identification by VITEK 2, and PCR confirmation	[45]
Saudi Arabia	2021	*Clostridium perfringens*	Minced meat	100	14.0	Culture-based detection, biochemical identification by VITEK 2	[46]
		*Clostridium difficile*			4.0		
Saudi Arabia	2021	Metallo-β-lactamases (MβLs) producing *Pseudomonas* spp.	Meat	45	55.0	Culture-based detection, biochemical identification	[47]
Saudi Arabia	2023	*Escherichia coli*	Carcass	40	100.0	Culture-based detection, biochemical identification, and PCR confirmation	[48]
			Meat cuts		70.0		
		*Salmonella* spp.	Carcass		40.0		
			Meat cuts		40.0		

**Table 2:** Overview of research on the chemical hazards of camel meat in some Middle Eastern countries published between 2014–2024.

Country	Year	Heavy Metal(s) ^a^	Pesticide /Drug Residue ^b^	Source(s)	Detection Method ^c^	Reference
Egypt	2015	Pb, Cd, Cu, Zn	-	Muscles, serum, lungs, liver, kidney	AAS	[51]
Egypt	2015	Pb, Cd, Cu, Al	Oxytetracycline	Muscles, kidney, and liver	Antibiotic residue detected by four-plate method using nutrient agar seeded with *Bacillus subtilis* and HPLC; Heavy metal detected by AAS	[52]
Egypt	2018	Pb, Cd, Hg	-	Muscles, kidney, and liver	AAS	[52]
Egypt	2019	-	Enrofloxacin, ciprofloxacin, tylosin, erythromycin, tetracycline, oxytetracycline, chlortetracycline, sulfamethazine and sulfaquinoxaline	Muscles, kidney, and liver	LC MS/MS	[53]
Iraq	2020	As, Cd, Pb, Cr, Cu, Fe, Mn, Zn, and Co	-	Muscles, kidney, and liver	ICP–OES	[54]
Iraq	2021	Pb, Cd, Zn, Cu, Co	-	Muscles, kidney, and liver	-	[55]
Jordan	2021	-	DDT and its metabolites, HCH and its isomers, Heptachlor and Heptachlor epoxide, Aldrin, Dieldrin, Endrin, and HCB	Meat, liver, milk	GC (HP 5890) equipped with Ni electron capture detector	[56]
Libya	2015	-	Oxytetracycline	Muscles, kidney, liver, and fat	LC MS	[57]
Morocco	2014	Cd, Pb	-	Liver, lung, meat, heart, and kidney	ICP–AES	[58]
Saudi Arabia	2015	-	DDT, DDE, DDD, DDA, Lindane, Dicofol	Muscles and liver of camels	GC/MS	[59]
Saudi Arabia	2017	As, Cd, Cr, Pb	-	Meat	Coupled plasma mass spectrometry	[60]
Saudi Arabia	2019	Cd, Pb, As	-	Muscles, kidney, and liver	ICP–OES	[61]
Saudi Arabia	2022	Zn, Fe, Cu, Pb, Cd	-	Tissues, muscles, kidney, and liver; serum and hair	AAS	[62]
Saudi Arabia	2023	Pb, Cd, Co	-	Meat, liver, young camels’ carcass tissues	ICP–OES equipped with a Meinhard Nebulizer type A^2^	[63]
Saudi Arabia	2024	As, Cr	-	Muscles, kidney, and liver	AAS used in conjunction with a (GFAAS)	[64]

^a^ Pb—Lead; Cd—Cadmium; Cu—Copper; Zn—Zinc; Al—Aluminum; Hg—Mercury; As—Arsenic; Cr—Chromium; Fe—Iron; Mn—Manganese; Zn—Zinc; Co—Cobalt; ^b^ DDT—Dichlordiphenyltrichloroethane; DDE—Dichlordiphenyldichloroethene; DDD—Dichlordiphenyldichloroethane; DDA—Bis 4 chlorophenyl acetic acid; HCH—Hexachlorocyclohexane; HCB—Hexachlorobenzene. ^c^ AAS—Atomic Absorption Spectrophotometer; LC MS/MS—Liquid Chromatography Tandem Mass Spectrometry; ICP–OES—Inductively Coupled Plasma–Optical Emission Spectrometry; GC—Gas chromatography; LC/MS—liquid chromatography mass spectrometry; ICP–AES—Inductively Coupled Plasma–Atomic Emission Spectroscopy; GC/MS—Gas Chromatography/Mass Spectrometry; GFAAS—Graphite Furnace Atomic Absorption Spectrometry.

**Table 3:** Overview of human cases of camel-origin zoonoses in some Middle Eastern countries published between 2014–2024.

Country	Year	Epidemiological Study	Camel-Origin Zoonotic Disease	No. of Human Cases	No. of Hospitalizations	No. of Deaths	Suspected Transmission Source(s)	Reference
Bahrain	2016	Outbreak investigations	MERS-CoV	1	1	ND	Direct contact	[71]
Oman	2017–2022	Outbreak investigations	MERS-CoV	18	18	ND	Direct contact	[71]
Qatar	2017–2022	Outbreak investigations	MERS-CoV	12	12	ND	Direct contact	[71]
United Arab Emirates	2017–2022	Outbreak investigations	MERS-CoV	14	14	0	Direct contact	[71]
Algeria	2014	Outbreak investigations	MERS-CoV	2	2	ND *	Direct contact	[72]
Egypt	2014	Outbreak investigations	MERS-CoV	1	1	ND	Direct contact	[72]
Kuwait	2014–2015	Outbreak investigations	MERS-CoV	4	4	ND	Direct contact	[72]
Oman	2014–2016	Outbreak investigations	MERS-CoV	8	8	ND	Direct contact	[72]
Qatar	2014–2016	Outbreak investigations	MERS-CoV	16	16	ND	Direct contact	[72]
Saudi Aribia	2016	Outbreak investigations	MERS-CoV	318	318	ND	Direct contact	[72]
	2015	Outbreak investigations	MERS-CoV	448	448	ND	Direct contact	
	2014	Outbreak investigations	MERS-CoV	566	566	ND	Direct contact	[72]
Egypt	2014–2015	Cross-sectional study	Trypanosomiasis	ND	ND	ND	Meat	[73]
Qatar	2015	Outbreak investigations	Brucellosis	14	14	ND	Unpasteurized camel milk	[74]
United Arab Emirates	2023–2024	Outbreak investigations	MERS-CoV	1	1	ND	Direct contact	[75]
Saudi Aribia	2023–2024	Outbreak investigations	MERS-CoV	5	5	ND	Direct contact	[75]
	2024	Outbreak investigations	MERS-CoV	3	3	1	Unknown	[76]
	2022–2023	Outbreak investigations	MERS-CoV	3	3	2	Direct contact	[77]
	2014–2015	Outbreak investigations	MERS-CoV	ND	190	69	Direct contact	[78]
United Arab Emirates	2016	Case study	Hepatitis E infection	ND	ND	ND	Meat	[79]

* ND, not determined.

## Data Availability

Not applicable.
